# 4-(Cyclo­propane­carboxamido)­benzoic acid

**DOI:** 10.1107/S1600536812038196

**Published:** 2012-09-29

**Authors:** Zhong-Qiang Sun, Zhen-Ya Ding, Zhi-Yu Shao

**Affiliations:** a College of Chemistry, Chemical Engineering and Biotechnology, Donghua University, Shanghai 201620, People’s Republic of China

## Abstract

In the title compound, C_11_H_11_NO_3_, the dihedral angle between the benzene ring and the cyclo­propane ring is 63.2 (1)°. In the crystal, mol­ecules are linked through classical cyclic carb­oxy­lic acid O—H⋯O hydrogen-bond inter­actions [graph set *R*_2_^2^(8)] giving centrosymmetric dimers which are extended along the *b*-axis direction through amide N—H⋯O hydrogen-bond inter­actions, giving one-dimensional ribbon structures. Weak C—H⋯O inter­actions are also present in the structure.

## Related literature

For general background to the biological activity of similar substituted benzoic acids, see: Gediya *et al.* (2008 ▶). For applications of analogs of the title compound, see: Sobotka *et al.* (1991 ▶); Chernoivanov *et al.* (1993 ▶, 1997 ▶). For graph-set analysis, see: Etter *et al.* (1990 ▶).
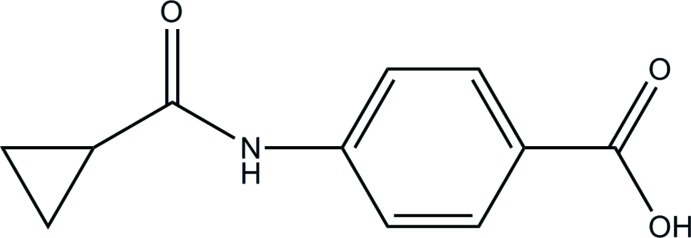


## Experimental

### 

#### Crystal data

C_11_H_11_NO_3_
*M* *_r_* = 205.21
Monoclinic, 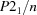

*a* = 13.2429 (14) Å
*b* = 4.7704 (5) Å
*c* = 16.7983 (18) Å
β = 111.227 (2)°
*V* = 989.21 (18) Å^3^
*Z* = 4
Mo *K*α radiation
μ = 0.10 mm^−1^
*T* = 293 K
0.31 × 0.21 × 0.17 mm

#### Data collection

Bruker SMART CCD area-detector diffractometer
Absorption correction: multi-scan (*SADABS*; Bruker, 2003 ▶) *T*_min_ = 0.789, *T*_max_ = 1.000
5558 measured reflections
1934 independent reflections
1682 reflections with *I* > 2σ(*I*)
*R*_int_ = 0.022

#### Refinement

*R*[*F* ^2^ > 2σ(*F* ^2^)] = 0.041
*wR*(*F* ^2^) = 0.119
*S* = 1.05
1934 reflections
145 parameters
1 restraint
H atoms treated by a mixture of independent and constrained refinement
Δρ_max_ = 0.15 e Å^−3^
Δρ_min_ = −0.20 e Å^−3^

### 

Data collection: *SMART* (Bruker, 2003 ▶); cell refinement: sAINT (Bruker, 2003 ▶); data reduction: *SAINT*; program(s) used to solve structure: *SHELXS97* (Sheldrick, 2008 ▶); program(s) used to refine structure: *SHELXL97* (Sheldrick, 2008 ▶); molecular graphics: *SHELXTL* (Sheldrick, 2008 ▶); software used to prepare material for publication: *SHELXTL*.

## Supplementary Material

Crystal structure: contains datablock(s) I, global. DOI: 10.1107/S1600536812038196/zs2222sup1.cif

Structure factors: contains datablock(s) I. DOI: 10.1107/S1600536812038196/zs2222Isup2.hkl

Supplementary material file. DOI: 10.1107/S1600536812038196/zs2222Isup3.cml

Additional supplementary materials:  crystallographic information; 3D view; checkCIF report

## Figures and Tables

**Table 1:** Hydrogen-bond geometry (Å, °)

*D*—H⋯*A*	*D*—H	H⋯*A*	*D*⋯*A*	*D*—H⋯*A*
N1—H1⋯O1^i^	0.827 (18)	2.144 (19)	2.9273 (16)	158.2 (16)
O3—H3⋯O2^ii^	0.87 (2)	1.80 (2)	2.6685 (15)	173 (3)
C2—H2⋯O1^i^	0.98	2.37	3.2034 (18)	142
C3—H3*B*⋯O2^iii^	0.97	2.54	3.350 (2)	141

Symmetry codes: (i) 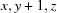; (ii) 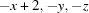; (iii) 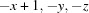.

**Fig. 1.**
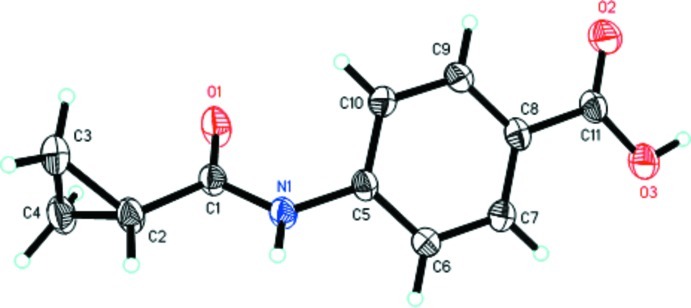
A view of the title compound with the atom numbering scheme. Displacement ellipsoids for non-H atoms are drawn at the 30% probability level.

**Fig. 2.**
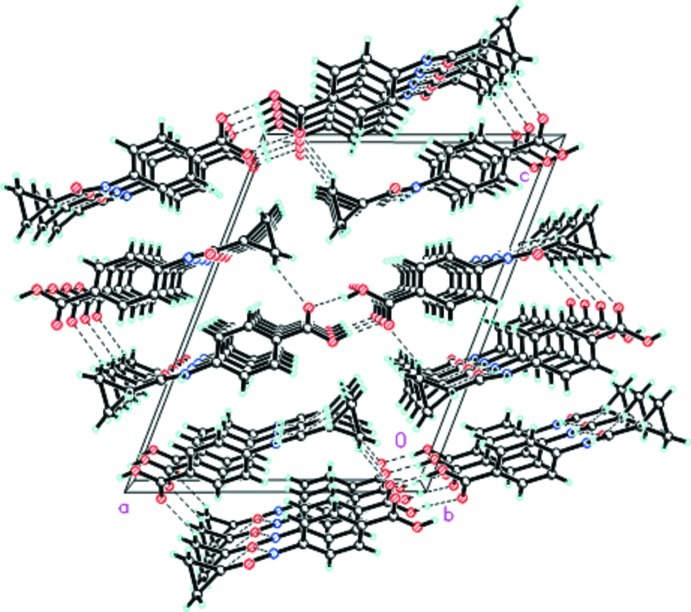
The structure of the infinite one-dimensional ribbon structures, with hydrogen bonds shown as dashed lines.

## supplementary crystallographic information

### Comment

The title compound, C_11_H_11_NO_3_, has been of great interest for many years because it has different biological activities and been used as a ligand in the synthesis of various Cathepsin-S reversible inhibitor compounds (Gediya *et al.*, 2008). In order to obtain a new potentially active histone deacetylase inhibitor, the title compound was synthesized and its crystal structure is reported here. In this molecule (Fig. 1), the dihedral angle between the benzene ring and the cyclopropane ring is 63.2 (1)°. In the crystal, molecules are linked through classic cyclic carboxylic acid O—H···O hydrogen-bonding interactions [graph set *R*^2^_2_(8) (Etter *et al.*, 1990)] giving centrosymmetric dimers which are extended along *b* through amide N—H···O hydrogen-bonding interactions (Table 1), giving one-dimensional ribbon structures (Fig. 2). Present also in the structure are weak C—H···O interactions to both amide and carboxyl O-acceptors.

### Experimental

Cyclopropanecarboxylic acid (500mg, 5.8 mmol) and N, N'- carbonyldiimidazole (1.035 g, 6.38 mmol) were dissolved in acetonitrile (5ml) and the solotion was stirred for 0.5h, then added dropwise into 5 ml of an acetonitrile solution of 4-aminobenzoic acid (1.59 g, 11.6 mmol). This solution was then added dropwise to 5 ml of a trifluoroacetic acid solution in acetonitrile (727 mg, 6.38 mmol) which was stirred for 1h. The resulting mixture was dried, then diluted with ethyl acetate, washed with water, then dried in vacuo. The residue was purified by colunm chromatography (CH_3_OH/CH_2_Cl_2_, 5/95). Crystals suitable for X-ray diffraction were grown in a dilute CH_3_OH/CH_2_Cl_2_ solution at room temperature by slow evaporation.

### Refinement

Hydrogen atoms on O and N were located in a difference-Fourier map and both coordinates and isotropic displacement parameters were refined. Other H-atoms were placed in idealized positions and allowed to ride on their respective parent atoms, with C—H = 0.93 Å (aromatic) or 0.97 or 0.98 Å (aliphatic) and *U*_iso_(H) = 1.2*U*_eq_(C).

### Crystal data

**Table:**

C_11_H_11_NO_3_	*F*(000) = 432
*M**_r_* = 205.21	*D*_x_ = 1.378 Mg m^−^^3^
Monoclinic, *P*2_1_/*n*	Mo *K*α radiation, λ = 0.71073 Å
Hall symbol: -P 2yn	Cell parameters from 2486 reflections
*a* = 13.2429 (14) Å	θ = 4.9–56.1°
*b* = 4.7704 (5) Å	µ = 0.10 mm^−^^1^
*c* = 16.7983 (18) Å	*T* = 293 K
β = 111.227 (2)°	Prismatic, colorless
*V* = 989.21 (18) Å^3^	0.31 × 0.21 × 0.17 mm
*Z* = 4	

### Data collection

**Table:**

Bruker SMART CCD area-detector diffractometer	1934 independent reflections
Radiation source: fine-focus sealed tube	1682 reflections with *I* > 2σ(*I*)
Graphite monochromator	*R*_int_ = 0.022
φ and ω scans	θ_max_ = 26.0°, θ_min_ = 2.4°
Absorption correction: multi-scan (*SADABS*; Bruker, 2003)	*h* = −16→16
*T*_min_ = 0.789, *T*_max_ = 1.000	*k* = −5→5
5558 measured reflections	*l* = −20→15

### Refinement

**Table:**

Refinement on *F*^2^	Secondary atom site location: difference Fourier map
Least-squares matrix: full	Hydrogen site location: inferred from neighbouring sites
*R*[*F*^2^ > 2σ(*F*^2^)] = 0.041	H atoms treated by a mixture of independent and constrained refinement
*wR*(*F*^2^) = 0.119	*w* = 1/[σ^2^(*F*_o_^2^) + (0.0639*P*)^2^ + 0.2147*P*] where *P* = (*F*_o_^2^ + 2*F*_c_^2^)/3
*S* = 1.05	(Δ/σ)_max_ < 0.001
1934 reflections	Δρ_max_ = 0.15 e Å^−^^3^
145 parameters	Δρ_min_ = −0.20 e Å^−^^3^
1 restraint	Extinction correction: *SHELXL97* (Sheldrick, 2008), Fc^*^=kFc[1+0.001xFc^2^λ^3^/sin(2θ)]^-1/4^
Primary atom site location: structure-invariant direct methods	Extinction coefficient: 0.022 (4)

### Special details

**Table:**

Geometry. All esds (except the esd in the dihedral angle between two l.s. planes) are estimated using the full covariance matrix. The cell esds are taken into account individually in the estimation of esds in distances, angles and torsion angles; correlations between esds in cell parameters are only used when they are defined by crystal symmetry. An approximate (isotropic) treatment of cell esds is used for estimating esds involving l.s. planes.
Refinement. Refinement of F^2^ against ALL reflections. The weighted R-factor wR and goodness of fit S are based on F^2^, conventional R-factors R are based on F, with F set to zero for negative F^2^. The threshold expression of F^2^ > 2sigma(F^2^) is used only for calculating R-factors(gt) etc. and is not relevant to the choice of reflections for refinement. R-factors based on F^2^ are statistically about twice as large as those based on F, and R- factors based on ALL data will be even larger.

### Fractional atomic coordinates and isotropic or equivalent isotropic displacement parameters (Å^2^)

**Table:**

	*x*	*y*	*z*	*U*_iso_*/*U*_eq_	
N1	0.56625 (9)	0.5748 (2)	0.15289 (8)	0.0404 (3)	
O1	0.49517 (9)	0.1514 (2)	0.16147 (9)	0.0605 (4)	
O2	0.87070 (9)	−0.0148 (3)	−0.01839 (8)	0.0707 (4)	
O3	0.98671 (9)	0.2842 (3)	0.06937 (8)	0.0647 (4)	
C1	0.49424 (11)	0.4057 (3)	0.16928 (9)	0.0394 (3)	
C2	0.41426 (12)	0.5484 (3)	0.19760 (11)	0.0498 (4)	
H2	0.4217	0.7520	0.2053	0.060*	
C3	0.30111 (13)	0.4331 (4)	0.16602 (12)	0.0649 (5)	
H3A	0.2418	0.5656	0.1533	0.078*	
H3B	0.2862	0.2707	0.1288	0.078*	
C4	0.36945 (14)	0.3933 (4)	0.25499 (11)	0.0591 (5)	
H4A	0.3969	0.2061	0.2730	0.071*	
H4B	0.3524	0.5010	0.2975	0.071*	
C5	0.64690 (10)	0.4785 (3)	0.12217 (8)	0.0361 (3)	
C6	0.75069 (11)	0.5867 (3)	0.15619 (10)	0.0466 (4)	
H6	0.7671	0.7235	0.1984	0.056*	
C7	0.83018 (11)	0.4915 (3)	0.12747 (9)	0.0479 (4)	
H7	0.8998	0.5649	0.1505	0.058*	
C8	0.80668 (10)	0.2874 (3)	0.06466 (9)	0.0407 (3)	
C9	0.70189 (11)	0.1846 (3)	0.02946 (9)	0.0471 (4)	
H9	0.6850	0.0505	−0.0136	0.057*	
C10	0.62238 (11)	0.2803 (3)	0.05789 (9)	0.0449 (4)	
H10	0.5522	0.2111	0.0337	0.054*	
C11	0.89105 (11)	0.1726 (3)	0.03496 (9)	0.0447 (4)	
H1	0.5640 (13)	0.744 (4)	0.1630 (10)	0.049 (5)*	
H3	1.0297 (19)	0.200 (5)	0.0483 (16)	0.110 (9)*	

### Atomic displacement parameters (Å^2^)

**Table:**

	*U*^11^	*U*^22^	*U*^33^	*U*^12^	*U*^13^	*U*^23^
N1	0.0449 (6)	0.0271 (6)	0.0596 (7)	0.0002 (5)	0.0314 (6)	−0.0046 (5)
O1	0.0739 (8)	0.0284 (5)	0.1042 (9)	0.0011 (5)	0.0621 (7)	−0.0012 (5)
O2	0.0470 (6)	0.0924 (10)	0.0813 (8)	−0.0001 (6)	0.0334 (6)	−0.0349 (7)
O3	0.0408 (6)	0.0872 (9)	0.0737 (8)	−0.0002 (6)	0.0300 (6)	−0.0204 (7)
C1	0.0437 (7)	0.0293 (7)	0.0531 (8)	0.0010 (5)	0.0270 (6)	−0.0004 (5)
C2	0.0569 (9)	0.0321 (7)	0.0781 (11)	−0.0027 (6)	0.0457 (8)	−0.0058 (7)
C3	0.0451 (8)	0.0789 (12)	0.0805 (12)	0.0013 (8)	0.0345 (8)	−0.0126 (10)
C4	0.0635 (10)	0.0607 (10)	0.0722 (11)	−0.0040 (8)	0.0474 (9)	−0.0034 (8)
C5	0.0384 (7)	0.0319 (6)	0.0445 (7)	0.0036 (5)	0.0226 (6)	0.0031 (5)
C6	0.0456 (8)	0.0478 (8)	0.0527 (8)	−0.0067 (6)	0.0252 (6)	−0.0136 (6)
C7	0.0357 (7)	0.0583 (9)	0.0534 (8)	−0.0053 (6)	0.0204 (6)	−0.0091 (7)
C8	0.0385 (7)	0.0473 (8)	0.0413 (7)	0.0064 (6)	0.0206 (6)	0.0030 (6)
C9	0.0435 (7)	0.0532 (9)	0.0499 (8)	−0.0001 (6)	0.0231 (6)	−0.0135 (7)
C10	0.0349 (7)	0.0503 (8)	0.0524 (8)	−0.0026 (6)	0.0193 (6)	−0.0098 (6)
C11	0.0391 (7)	0.0559 (9)	0.0434 (7)	0.0063 (6)	0.0199 (6)	0.0008 (7)

### Geometric parameters (Å, º)

**Table:**

N1—C1	1.3515 (17)	C4—H4A	0.9700
N1—C5	1.4202 (16)	C4—H4B	0.9700
N1—H1	0.827 (18)	C5—C10	1.3827 (19)
O1—C1	1.2208 (17)	C5—C6	1.3832 (19)
O2—C11	1.2249 (19)	C6—C7	1.3839 (19)
O3—C11	1.3007 (18)	C6—H6	0.9300
O3—H3	0.870 (17)	C7—C8	1.386 (2)
C1—C2	1.4753 (18)	C7—H7	0.9300
C2—C4	1.498 (2)	C8—C9	1.386 (2)
C2—C3	1.501 (2)	C8—C11	1.4834 (18)
C2—H2	0.9800	C9—C10	1.3817 (19)
C3—C4	1.452 (3)	C9—H9	0.9300
C3—H3A	0.9700	C10—H10	0.9300
C3—H3B	0.9700		
			
C1—N1—C5	124.04 (11)	C2—C4—H4B	117.7
C1—N1—H1	117.4 (11)	H4A—C4—H4B	114.8
C5—N1—H1	118.6 (11)	C10—C5—C6	119.67 (12)
C11—O3—H3	107.8 (18)	C10—C5—N1	120.68 (12)
O1—C1—N1	122.42 (12)	C6—C5—N1	119.65 (12)
O1—C1—C2	121.98 (12)	C5—C6—C7	120.07 (13)
N1—C1—C2	115.59 (12)	C5—C6—H6	120.0
C1—C2—C4	118.53 (13)	C7—C6—H6	120.0
C1—C2—C3	117.27 (13)	C6—C7—C8	120.44 (13)
C4—C2—C3	57.93 (11)	C6—C7—H7	119.8
C1—C2—H2	116.7	C8—C7—H7	119.8
C4—C2—H2	116.7	C7—C8—C9	119.16 (12)
C3—C2—H2	116.7	C7—C8—C11	121.77 (13)
C4—C3—C2	60.91 (11)	C9—C8—C11	119.06 (13)
C4—C3—H3A	117.7	C10—C9—C8	120.42 (13)
C2—C3—H3A	117.7	C10—C9—H9	119.8
C4—C3—H3B	117.7	C8—C9—H9	119.8
C2—C3—H3B	117.7	C9—C10—C5	120.19 (13)
H3A—C3—H3B	114.8	C9—C10—H10	119.9
C3—C4—C2	61.15 (11)	C5—C10—H10	119.9
C3—C4—H4A	117.7	O2—C11—O3	123.00 (13)
C2—C4—H4A	117.7	O2—C11—C8	121.50 (13)
C3—C4—H4B	117.7	O3—C11—C8	115.50 (13)
			
C5—N1—C1—O1	2.5 (2)	C5—C6—C7—C8	−0.1 (2)
C5—N1—C1—C2	−177.69 (12)	C6—C7—C8—C9	1.6 (2)
O1—C1—C2—C4	28.8 (2)	C6—C7—C8—C11	−177.50 (14)
N1—C1—C2—C4	−150.99 (15)	C7—C8—C9—C10	−1.3 (2)
O1—C1—C2—C3	−37.6 (2)	C11—C8—C9—C10	177.75 (13)
N1—C1—C2—C3	142.56 (15)	C8—C9—C10—C5	−0.4 (2)
C1—C2—C3—C4	108.12 (16)	C6—C5—C10—C9	1.9 (2)
C1—C2—C4—C3	−105.96 (17)	N1—C5—C10—C9	−178.93 (14)
C1—N1—C5—C10	43.3 (2)	C7—C8—C11—O2	177.35 (16)
C1—N1—C5—C6	−137.46 (15)	C9—C8—C11—O2	−1.7 (2)
C10—C5—C6—C7	−1.6 (2)	C7—C8—C11—O3	−2.6 (2)
N1—C5—C6—C7	179.14 (13)	C9—C8—C11—O3	178.37 (13)

### Hydrogen-bond geometry (Å, º)

**Table:**

*D*—H···*A*	*D*—H	H···*A*	*D*···*A*	*D*—H···*A*
N1—H1···O1^i^	0.827 (18)	2.144 (19)	2.9273 (16)	158.2 (16)
O3—H3···O2^ii^	0.87 (2)	1.80 (2)	2.6685 (15)	173 (3)
C2—H2···O1^i^	0.98	2.37	3.2034 (18)	142
C3—H3*B*···O2^iii^	0.97	2.54	3.350 (2)	141

Symmetry codes: (i) *x*, *y*+1, *z*; (ii) −*x*+2, −*y*, −*z*; (iii) −*x*+1, −*y*, −*z*.
